# A study of the relationship between circulating cytokines (interleukin-2 receptor and tumor necrosis factor receptor 2) and risk of B-cell non-hodgkin lymphoma

**DOI:** 10.1007/s00277-024-05996-2

**Published:** 2024-09-24

**Authors:** Sara El-Sayed Abd El-Ghani, Heba Youssef Abido, Nehad Mohamed Tawfik, Gehan Shaheen, Hend Nabil Ellithy

**Affiliations:** 1 Internal Medicine Department, Clinical Hematology Unit, Kasr Alainy Faculty of Medicine, Cairo University, Kasr Al-Ainy, old Cairo, Cairo, Egypt; 2 Department of Clinical and Chemical Pathology, Kasr Alainy Faculty of Medicine, Cairo University, Cairo, Egypt

**Keywords:** B-NHL, Prognosis, Cytokines, sIL-2R, sTNF-R2

## Abstract

Mature B-cell non-Hodgkin lymphoma (B-NHL) occurs due to uncontrolled B-lymphocyte clonal expansion. Cytokines can directly stimulate B-cell proliferation and prevent B-cell apoptosis. Dysregulation of cytokines may play an important role in the development of B-NHL by enhancing chromosomal translocation, which is the hallmark of B-NHL. Both interleukin 2 and tumor necrosis factor-α are proinflammatory cytokines and play important roles in the growth, differentiation, and apoptosis of B cells.

We conducted a prospective case-control study applied to 50 patients with B-NHL at Kasr Al Aini Hospital, Cairo University, and 50 age- and sex-matched controls. Clinical, laboratory and imaging data were collected. In all patients and controls, sIL-2R and sTNF-R2 levels were measured by enzyme-linked immunosorbent assay (ELISA). The Spearman correlation test was used to evaluate the correlation between the studied cytokines and clinical, laboratory and imaging findings. Sensitivity analysis was conducted to detect the cutoff values of the studied cytokines.

Serum levels of sIL-2R and sTNF-R2 were significantly higher in patients than in controls. Additionally, their levels were significantly higher in aggressive types and advanced stages of lymphoma. Also, the studied cytokines were significantly correlated with different clinical and laboratory parameters of lymphoma. The level of sIL-2R and sTNF-R2 were closely related to the type of lymphoma (*P* value ˂ 0.001 and 0.012, respectively), further it was also associated with the natural history of lymphoma (aggressive vs. indolent) (*P* value ˂0.001 and 0.04 respectively).

We concluded that Pretreatment levels of sIL-2R and sTNF-R2 may play a role in the natural history and prognosis of lymphoma. They may be used as a prognostic factor for B-NHL patients and may also help with treatment decisions.

**Supplementary Information:**

The online version contains supplementary material available at 10.1007/s00277-024-05996-2.

## Background

Mature B-cell non-Hodgkin lymphoma (B-NHL) is a heterogeneous group of malignancies involving the uncontrolled clonal expansion of transformed B cells. It represents approximately 83% of all NHLs [1]. The etiology of B-NHL remains unknown, but the most consistent risk factors are altered immunity conditions. Immunodeficiency is a vital risk factor for NHL [2]. Autoimmune conditions, such as rheumatoid arthritis, systemic lupus erythematosus, and Sjögren’s disease, are associated with an increased risk of B-NHL [3]. In contrast, atopic conditions, such as asthma, hay fever, and eczema, may be associated with a decreased risk of B-NHL [4]. Chronic antigenic stimulation by Epstein–Barr virus (EBV), *H. pylori* or hepatitis C virus is also associated with increased B-NHL risk [5]. 

A common characteristic of these conditions is the dysregulation of cytokines, which play essential roles in immune cell development and immune functions [6]. In addition, cytokines can directly stimulate B-cell proliferation, prevent B-cell apoptosis, and promote B-cell variable joining and diversity V(D)J gene segment recombination and isotype switching, which collectively enhance the likelihood of chromosome translocations, which are a hallmark of B-NHL [7]. Translocations can activate protooncogenes such as C-myc and/or inactivate tumor suppressor genes, which may ultimately lead to the malignant transformation of B cells [8]. Hence, it can be hypothesized that cytokines play a crucial role in B-cell lymphomagenesis. IL-2 is a proinflammatory cytokine, and activated B cells proliferate in response to the binding of IL-2 to cellular receptors [9]. 

Interleukin 2 receptor alpha (sIL-2R) and tumor necrosis factor receptor II (sTNF-R2) are produced by cells of the immune system, including dendritic cells, macrophages and monocytes and B cells. Also T cells produce notable amounts of sIL-2R after activation, with the exception of regulatory T cells which do not require (in vitro) activation [10, 11]. 

The primary function of IL-2 is to generate a cytotoxic immune response by promoting natural killer (NK) and lymphokine-activated killer (LAK) cells, which are important in the immune surveillance of potentially malignant cells [12]. Soluble IL-2R (sIL-2R), released from the cell membrane by cleavage of IL-2R, binds free IL-2 and inhibits its tumor surveillance function, which may explain its positive association with B-NHL [9]. 

TNF-α has strong proinflammatory effects and can induce the production of other proinflammatory cytokines, such as IL-1, IL-6, and IL-8. TNF-α also plays essential roles in mediating B cells’ activation, growth, differentiation, apoptosis, and migration [8]. Although serum Tumor Necrosis Factor Receptor 2 (sTNF-R2) inhibits the biological activity of TNF-α at high concentrations, it binds with TNF-α. It protects it from breakdown, thus acting as a slow-release reservoir. It has been increasingly used as a reliable surrogate for TNF-α since it has a longer half-life and can be measured quickly and reliably in frozen blood samples [9]. 

In this study, we aimed to determine whether elevated serum levels of interleukin 2 receptor alpha (sIL-2R) and tumor necrosis factor receptor II (sTNF-R2) may be a risk factor for NHL in Egyptians. In addition, if the elevation of these cytokines is linked to certain types of NHL. We also aimed to assess whether the elevation of these cytokines may be of prognostic value.

## Methods

### Study design

This prospective case-control study included 50 patients with B-non-Hodgkin lymphoma attending the outpatient clinic of Kasr El-Aini Hospital, Faculty of Medicine, Cairo University, and 50 controls of the same age and sex. **Sample size calculation** was based on a previous study by Bien & Balcerska, which reported a large effect size [13]. So, we needed to study 42 participants per group, which was compensated by 15% due to the use of nonparametric tests, so the final sample size was **50** subjects per group (total sample size 100) to be able to reject the null hypothesis that the population means sIL-2R level of the study groups are equal with probability (power) 0.95. The Type I error probability associated with the test of this null hypothesis is 0.05. The sample size was calculated using the G power program (version 3.1.9.2) [14]. 

**Inclusion criteria** included recently diagnosed B-NHL patients aged ˃16 years who had not started receiving chemotherapy for B-NHL at the time of recruitment and were not diagnosed with any autoimmune disease. Cases were diagnosed through clinical assessment and investigations, mainly tissue biopsy (lymph node biopsy and bone marrow biopsy), in addition to other laboratory investigations and imaging studies.

**Exclusion criteria** included patients whose age ˂ 16 years, patients with other types of NHL rather than B-NHL and patients with B-NHL who were receiving chemotherapy. All our controls fulfilled the inclusion criteria to be healthy individuals, not having any evidence of infection or chronic inflammatory condition, and not having a chronic illness.

### Data collection

The included patients provided a full medical history, including age, sex, residency, symptoms, especially anaemic manifestations, bleeding tendency, recurrent infections, and B symptoms (night fever, night sweats, loss of weight and loss of appetite). A thorough clinical examination assessed pallor, jaundice, ecchymosis, lymph node enlargement, and hepatosplenomegaly.

Laboratory assessments, including complete blood count (CBC), reticulocyte count, lactate dehydrogenase (LDH) and beta-2-microglobulin (β2 M), were assessed for the included patients. A bone marrow examination assessed our patients’ percentage and pattern of atypical cell infiltration. sIL-2R and sTNF-R2 levels were measured in all patients.

### Sampling

Venous blood samples (5 ml) from each patient were withdrawn into a red top vacutainer tube for serum samples, allowed to clot for 30 min at room temperature before centrifugation and then centrifuged rapidly at 1000 x g for 15 min. The serum obtained was aliquoted and stored at ≤ -20 °C until used for sIL-2R and sTNF-R2.

### Measurement of plasma sIL-2R and sTNF-R2

This was done using the Human Interleukin 2 Receptor alpha (sIL-2R) enzyme-linked immunosorbent assay (ELISA) Quantikine™ Kit for the quantitative determination of human Interleukin 2 Receptor alpha (sIL-2R) concentrations in serum, catalog numbers DR2A00- SR2A00 - PDR2A00 (R&D Systems, Inc., Bio-Techne Ltd., USA) [15], and the Quantikine™ ELISA Human TNF RII/TNFRSF1B Immunoassay Kit for the quantitative determination of human Tumor Necrosis Factor Receptor II (sTNF-R2) concentrations in serum, catalog numbers DRT200 - SRT200 - PDRT200 (R&D Systems, Inc., Bio-Techne Ltd., USA) [16]. This sandwich kit is for the accurate in vitro quantitative detection of plasma sIL-2R and sTNF-R2.

#### Imaging

Abdominal ultrasound to assess the size of the spleen in centimeters and computed tomography (CT) of the neck, chest and abdomen to assess the number and size of enlarged lymph nodes were assessed for all included patients.

The International Prognostic Index (IPI) was assessed for all patients [17]. 

#### Confidentiality of data

After explaining the nature of the study, informed consent was obtained from every patient. There are no conflicts of interest regarding this research. Every patient had a code number, and symbols corresponding to their name and address were kept in a special file. The results were used only in scientific publications.

#### Measures to alleviate risk

Samples were obtained under complete aseptic conditions. Any unexpected risks were documented and declared to the participants and the ethical committee.

#### Ethical consideration

The ethical committee of the Faculty of Medicine, Cairo University, approved the study, which was performed in accordance with the principles of the Declaration of Helsinki.

### Statistical analysis

Data were coded and entered using the statistical package SPSS version 22. Data were summarized using the mean and standard deviation for normally distributed variables, the median and interquartile range for quantitative variables that were not normally distributed and the number and percent for qualitative variables.

Comparisons between groups were performed using the nonparametric Mann‒Whitney test for quantitative variables that were not normally distributed [18] and the Chi-square test for qualitative variables [19]. Correlations were performed to test for linear relations between variables. ROC curve analysis was performed to test the ability of a variable to discriminate between cases and controls and to find the best cutoff value. *P* values < 0.05 were considered statistically significant [20]. 

## Results

### Demographics and clinical characteristics of the study population

This study included 50 patients diagnosed with lymphoma and 50 healthy controls. Forty-two per cent of the patients were females, and 58% were males. The patients’ ages ranged between 16 and 77 years. The mean was 54.3, and the standard deviation (SD) was +/-12.6.

According to the diagnosis, 28/50 patients (56%) were diagnosed with diffuse large B-cell lymphoma (DLBCL), 5/50 (10%) were diagnosed with follicular lymphoma (FL), and 17/50 (34%) were diagnosed with chronic lymphocytic leukemia (CLL). Fifty-six per cent of the patients had aggressive lymphoma (DLBCL), and 44% of the patients had indolent lymphoma (FL + CLL). Regarding the IPI scoring, 3 patients were IPI-1 (6%), 6 patients were IPI-2 (12%), 14 patients were IPI-3 (28%), 13 patients were IPI-4 (26%) and 14 patients were IPI-5 (28%). Using the Ann Arbor staging, most of the patients 39/50 (78%) were stage IV, 7/50 (14%) were stage III and 4/50 (8%) were stage II. **(**Table 1**)**

**Table 1:** IL2R and TGFR in patients and controls

Parameter	Patients (*n* = 50)	Controls (*n* = 50)	*P* value
**IL2R (pg/mL)**	Median = 2404.5 Interquartile range (IQR): (1119: 5652)	Median = 158.5 IQR: (112: 287)	˂0.001
**TGFRII (pg/mL)**	Median = 200 IQR: (58: 319)	Median = 16 IQR: (9: 24)	˂0.001

### Laboratory findings and imaging of the studied population

Analysis of the patients’ and controls’ results revealed that sIL-2R and sTNF-R2 are significantly higher in patients with lymphoma than in controls (*P* value ˂ 0.001 and ˂ 0.001, respectively). (Table 2**)**

**Table 2:** The relation between the clinical parameters and the studied biomarkers (serum TNFRII and IL2R)

Clinical parameter		TNFRII				IL2R			
		Median	1st quartile	3rd quartile	*P* value	Median	1st quartile	3rd quartile	*P* value
**Anemic manifestation**	Present *N* = 31	241	129	417	0.001	4263	2463	6992	˂0.001
	Absent *N* = 19	59	38	134		781	411	1628	
**Bleeding tendency**	Present *N* = 10	385	237.75	529.75	0.005	6566.5	4685.25	7742	0.001
	Absent *N* = 40	130	53.25	272.75		1732.5	809.75	3511.5	
**Weight loss**	Present *N* = 35	239	126	359	0.003	3459	1653	6981	0.001
	Absent *N* = 15	51	29	242		1227	411	1628	
**Fever**	Present *N* = 41	239	81.50	348.5	0.004	3147	1217.5	6499	0.001
	Absent *N* = 9	39	25	75.5		1099	277.5	1302	
**Splenomegaly**	Present *N* = 40	238.5	70.25	357	0.015	3302.5	1401	6631	˂0.001
	Absent *N* = 10	56	44.75	160.25		455.5	384.5	1177.5	
**Pattern of BM infiltration**	Diffuse *N* = 16	312.5	231.25	521.25	0.02	6566.5	3844.75	7863	˂0.001
	Nodular *N* = 5	59	53	232		3458	1831.5	5296.5	
	Patchy *N* = 18	132.5	53	262		1463.5	701.5	2596.75	
	No infiltration *N* = 11	69	36	126		1119	278	1628	
**Stage of the disease**	II *N* = 4	32	89	71	0.026	347.5	108.5	1325.25	0.007
	III *N* = 7	24.5	54	472		1287	411	2346	
	1 V *N* = 39	239	59	347		3147	1227	6235	

The patients were divided into 4 groups according to the age, both markers were significantly different between the 4 groups. On comparing the four groups regarding the studied markers, we found that sTNF-R2 showed a statistically significant difference only between group 1 (Q1) and group 4 (Q4), *P* value = 0.002. The sIL-2R showed a statistically significant difference only between group 1 (Q1) and group 3 (Q3). Regarding gender, there was no statistically significant difference between males and females regarding both markers. (Table 3)

**Table 3:** The relation between the quartiles of age and sex of the patients and the studied biomarkers (serum TNFRII and IL2R)

	sTNFRII mean +/- SD	*P* value	sIL2R	*P* value
**Age**				
Q1	82.33 +/-89.00	0.004	1818.08+/-2291.47	0.004
Q2	300.38+/-472.38		2117.38+/-1798.61	
Q3	220.83+/-105.78		4644.58+/-2244.31	
Q4	341.38+/-198.83		4512.00+/-3236.27	
**Gender**				
Male	263.55+/- 337.23	0.945	3391.83+/- 2847.80	0.761
Female	206.57 +/ -176.39		3112.90+/- 2597.44	

sTNF-R2 and sIL-2R were significantly higher in patients who experienced anemia, bleeding tendency, weight loss, fever and splenomegaly compared to those who did not (*P* values were 0.001 and ˂0.001; 0.005 and 0.001; 0.003 and 0.001; 0.004 and 0.001;0.015 and ˂0.001 respectively). The serum level of sIL-2R was significantly different (*P* value =˂0.001) according to the pattern of bone marrow infiltration; the highest level was in diffuse pattern (7863 pg/ml), followed by nodular pattern (5296.5 pg/ml), and patchy pattern ( 2596.75 pg/ml), the lowest level was in patients with no bone marrow infiltration (1628 pg/ml). The serum level of sIL-2R was significantly higher in advanced stages of lymphoma compared to early stages (*P* value = 0.007) as the median was 347.5, 1287 and 3147 in stages II, III and IV, respectively. (Table 4**)**

**Table 4:** The association between laboratory and imaging findings and the studied biomarkers (serum TNFRII and IL2R)

Variable	IL2R		TGFR	
	*R*	*P* value	*R*	*P* value
Hemoglobin	- 0.720	˂0.001	- -0.543	˂0.001
Platelets	-0.564	˂0.001	-0.299	0.035
LDH	-0.664	˂0.001	0.485	˂0.001
B2 Microglobulin	0.408	0.003	0.345	0.014
Spleen size	0.705	˂0.001	0.478	˂0.001
BM infiltration	0.764	˂0.001	0.593	˂0.001
Extent of LN infiltration by CT	0.488	˂0.001	0.393	0.005
IPI score	0.752	˂0.001	0.609	˂0.001

Laboratory and imaging parameters were significantly correlated with the studied biomarkers; the serum level of sIL-2R and sTNF-R2 were significantly correlated with hemoglobin level, platelets count, LDH, B2M, Spleen size, BM infiltration, Extent of LN infiltration by CT and IPI score (*P* value = ˂0.001 and ˂0.001, ˂0.001 and 0.035, ˂0.001 and ˂0.001, 0.003 and 0.014, ˂0.001 and ˂0.001, ˂0.001 and ˂0.001, ˂0.001 and 0.005, ˂0.001 and ˂0.001 respectively). **(**Table 5**)**

**Table 5:** The relation between serum levels of the studied biomarkers (TNFRII and IL2R) and the different types of lymphoma among the studied patients’ group

Type of Lymphoma	IL2R (pg/mL) Median	*P* value	TNFRII (pg/mL) Median	*P* value
**DLBCL (** *n* ** = 28)**	5109	˂0.001	244.5	0.012
**CLL (** *n* ** = 17)**	1161		69	
**FL (** *n* ** = 5)**	896		42	
**Aggressive lymphoma** **(** *n* **= 28)**	5109	˂0.001	244.5	0.04
**Indolent lymphoma** **(** *n* **= 22)**	1028.5		64	

### **Relation between measured sIL-2R and sTNF-R2 and the type of lymphoma in the study**

The studied biomarkers were compared to the type of lymphoma and to whether it was indolent or aggressive; sIL-2R and sTNF-R2 serum levels were significantly different among the different types of lymphoma (*P* value ˂ 0.001 and 0.012, respectively); they were the highest in DLBCL followed by CLL then FL. Further, when they were compared to the type either aggressive (DLBCL) or indolent (FL + CLL), they were also significantly different (*P* value ˂0.001 and 0.04 respectively). **(**Table 5**)**

**Fig. 1:**
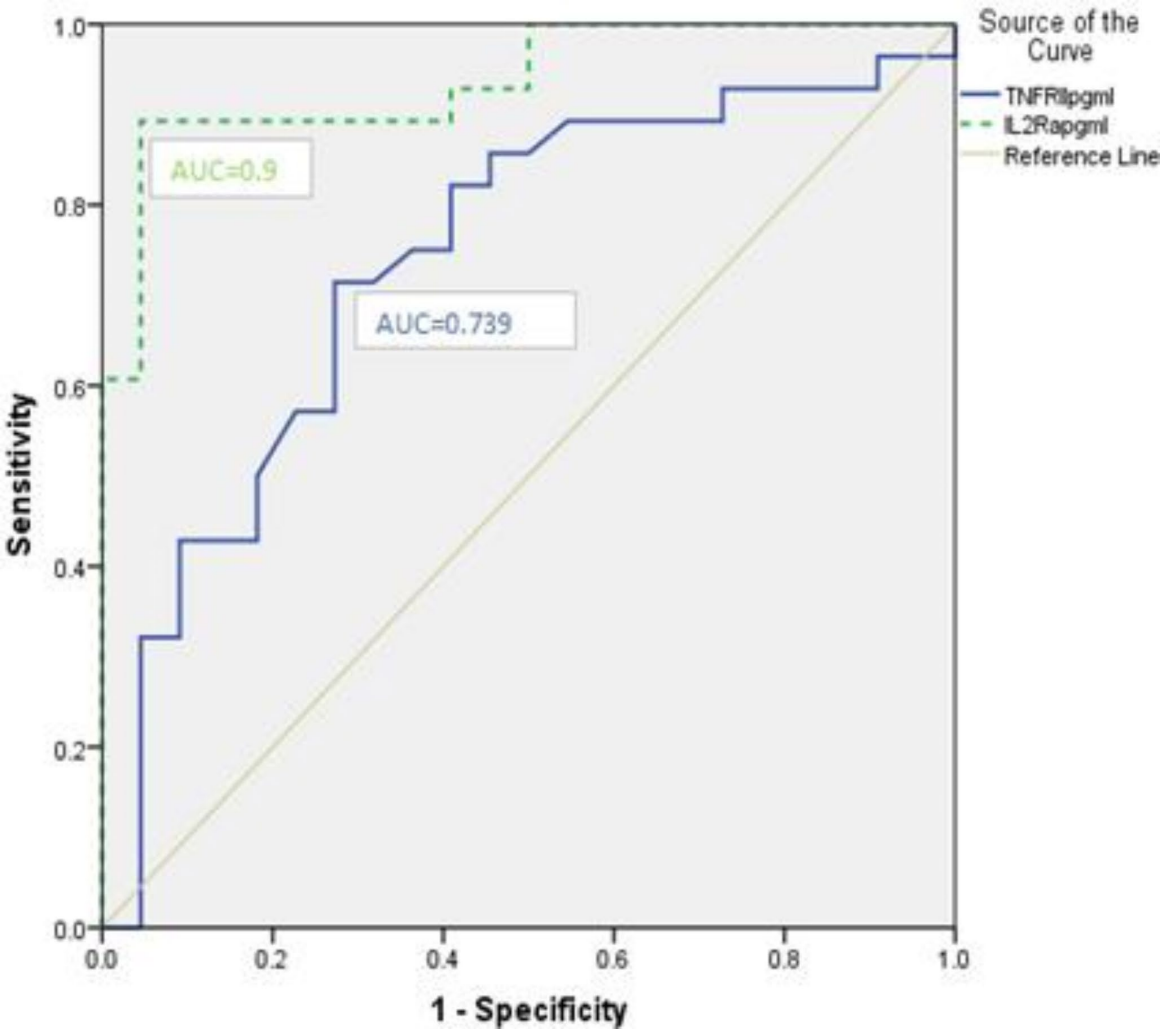
ROC curves for sTNF-R2 and sIL-2R. Based on ROC curves, the optimal cut-off value for sTNF-R2 is 153 pg/mL AUC = 0.739 and for sIL-2R is 2034.5 pg/ml, AUC = 0.937

Based on the ROC curves, the optimal cutoff value for sIL-2R was 2304 pg/mL, with a sensitivity of 89.3% and a specificity of 95.5%. For sTNF-R2, the optimal cutoff value was 153 pg/mL, with a sensitivity of 89.3% and a specificity of 72.7%. These cutoff values were closely related to the classification of the type of lymphoma; the sIL-2R cutoff value of 2034.5 pg/ml could detect 25/28 (89.3%) as aggressive lymphoma and 21/22 (95.5%) as indolent lymphoma. The sTNF-R2 cutoff value of 153 pg/mL could detect 20/28 (71.4%) as aggressive lymphoma and 16/22 (72.7%) as indolent lymphoma. **(**Fig. 1**)**

## Discussion

In our study, we found that sIL-2R and sTNF-R2 were significantly higher in patients with lymphoma compared to controls. This confirms that cytokine dysregulation may play an important role in the development of B-NHL. Additionally, their levels were higher in aggressive types and advanced stages of lymphoma, which may suggest their role in B-NHL prognosis and identification.

Upon comparing some of the laboratory and pathological parameters of our cases to those of other previous studies, Sadia et al., similar to our results, found that diffuse bone marrow infiltration was the most common [21]. However, Ayaz et al. disagreed with our results and found that interstitial infiltration was the most common pattern [22]. Naghmana et al., similar to our results, found that serum β2 microglobulin and LD2 levels significantly increased in NHL patients compared to controls [23]. 

Our results agree with Gu et al.‘s case-control study who measured fifteen cytokines in 92 cases with B-NHL and 2 matched controls per case. They found that serum levels of certain cytokines, including sIL-2R, IL-5, IL-13, TNF-α, and sTNF-R2, were associated with the risk of B-NHL [24]. Makgoeng et al. examined the associations between prediagnosis tumor necrosis factor α (TNFα) and the risk of NHL. They found that elevated circulating levels of TNF-α were consistently associated with an increased risk of NHL, suggesting the potential utility of these biomarkers in population risk stratification and prediction [25]. 

Unlike our results, M et al. conducted a case-control analysis (272 cases and 541 matched controls) for measuring a 10-plex panel of cytokines. They found that IL-10 was the only cytokine associated with NHL, TNF-α and IL-8 showed borderline elevated risks, whereas IFN-γ, IL-1β, IL-2, IL-4, IL-5, IL-6, and C-reactive protein (CRP) were not associated with NHL [26]. 

In our study, patients with bone marrow infiltration showed significantly higher levels of the studied inflammatory markers (sIL-2R and sTNF-R2) than those with no bone marrow infiltration, which may suggest that these inflammatory markers may contribute to the occurrence of bone marrow infiltration in lymphoma patients. Additionally, in patients with bone marrow infiltration, there is a direct relationship between the levels of sIL-2R and sTNF-R2 and the pattern of bone marrow infiltration. This finding also may suggest that these inflammatory markers may contribute to the occurrence of bone marrow infiltration and the severity of bone marrow involvement.

These results agree with the results of Kusano et al., who detected serum levels of sIL-2R in pretreated FL cases and found that stage III/IV, nodal lesions, bone marrow involvement, bulky tumor, elevated LDH, increased β2 M, high Follicular Lymphoma International Prognostic Index (*FLIPI*) and high FLIPI2 were significantly associated with high sIL-2R levels [27]. 

We found that the serum level of sIL-2R and sTNF-R2 was significantly higher in advanced stages of lymphoma according to the Ann Arbor stage staging system. Zhong et al. investigated the association of pretreatment inflammatory status with survival time and developed a prognostic nomogram incorporating inflammatory cytokines in 886 NHL. Their results showed that increased levels of LDH, β2-M, CRP, IL-2R, IL-6, IL-10 and TNF-α were closely related to advanced Ann Arbor stage and poor performance status [28]. Increased levels of sIL-2R were observed in patients with bone marrow or liver involvement, and increased levels of TNF-α were observed in patients with lung involvement.

In the studied group, we found that the higher the patients’ international prognostic index (IPI) was, the higher the serum levels of the studied inflammatory markers (sIL-2R and sTNF-R2). These inflammatory markers may help predict the prognosis of lymphoma patients. This result agreed with Elizabeth et al., who assessed the prognostic value of a large panel of cytokines in aggressive NHL and compared it to the parameters of the IPI. Their results were correlated with complete remission (CR), overall survival (OS) and failure-free survival (FFS). They concluded that sIL-2R and IL-6 serum levels are elevated in high-grade NHL and are correlated with CR, OS and FFS, but this study did not support their independent prognostic value. However, sIL-2R and IL-6 measurements may improve risk assignment by IPI and allow a better prognostic evaluation of patients with intermediate prognosis NHL [29]. 

Our results showed that the more advanced the stage of B-NHL, the higher the levels of sIL-2R and sTNF-R2. This suggests the role of these inflammatory cytokines in detecting the prognosis of lymphoma patients. The serum levels of the studied inflammatory cytokines (sIL-2R and sTNF-R2) in different types of lymphoma were variable. The highest level of both was recorded in DLBCL, followed by CLL, and the lowest level was in FL. The serum levels of the studied inflammatory cytokines (sIL-2R and sTNF-R2) were higher in aggressive lymphoma than in indolent lymphoma. This result agreed with Yoshida et al., who performed a study to detect the clinical significance of sIL-2R levels in B-cell lymphomas. They retrospectively analyzed 104 DLBCL and 54 FL cases. In DLBCL, patients with high sIL-2R had poor prognosis when compared to patients with low sIL-2R. Patients with high sIL-2R in FL tended to have poor prognosis, although the difference did not reach significance. Furthermore, no FL patients with low sIL-2R died. Thus, sIL-2R is a useful prognostic factor in DLBCL and in FL [30]. 

The limitations of our study were the small patient sample and the absence of serial samples of both sIL-2R and sTNF-R2 from patients after starting chemotherapy.

## Conclusion

We concluded that sIL-2R and sTNF-R2 may be a risk factor for B-NHL, as their serum levels were significantly higher in patients compared to the control group. This confirms that cytokine dysregulation may play an important role in the development of B-NHL. Also, we found that abnormal pretreatment serum levels of the studied inflammatory cytokines (sIL-2R and sTNF-R2) were associated with poor prognosis. Upon further validation, the prognostic role of sIL-2R and sTNF-R2 may help the treatment decision.

### Recommendations

We recommend that larger studies be conducted on patients with B-NHL to study the prognostic role of sIL-2R and sTNF-R2 further. Additionally, we recommend measuring serum levels of sIL-2R and sTNF-R2 pretreatment, serially during treatment, and posttreatment to determine their burden of disease and correlate their response to treatment with their serum levels.

## Electronic supplementary material

Below is the link to the electronic supplementary material.

Supplementary Material 1

Supplementary Material 2

## Data Availability

No datasets were generated or analysed during the current study.

## Abbreviations

B-NHL: B-cell non-Hodgkin lymphoma
CBC: Complete blood count
CLL: Chronic lymphocytic leukemia
CR: Complete remission
CRP: C-reactive protein
CT: Computed tomography
DLBCL: Diffuse large B-cell lymphoma
EBV: Epstein–Barr virus
ELISA: Enzyme-linked immunosorbent assay
FFS: Failure free survival
FL: Follicular lymphoma
FLIPI: Follicular Lymphoma Interntional Prognostic Index
IL: Interleukin
IL-2R α (IL-2Ra): Interleukin 2 Receptor alpha
IPI: International prognostic index
LAK: Lymphokine-activated killer
LD2: Lactate dehydrogenase 2
LDH: Lactate dehydrogenase
NHL: Non-Hodgkin lymphoma
NK: Natural killer
OS: Overall survival
SD: Standard deviation
sIL-2R: soluble Interleukin 2 Receptor alpha
SPSS: Statistical Package for the Social Sciences
sTNF-R2: Serum Tumor Necrosis Factor Receptor II
TNF-RII: Tumor Necrosis Factor Receptor II
V(D)J: Joining and diversity
β2 M: β2 microglobulin
