# Supplementary figures and images for: A study of the relationship between circulating cytokines (interleukin-2 receptor and tumor necrosis factor receptor 2) and risk of B-cell non-hodgkin lymphoma
Source: Ann Hematol. 2024 Sep 24;104(1):665–73. doi: 10.1007/s00277-024-05996-2 (PMC11868312; doi:10.1007/s00277-024-05996-2)

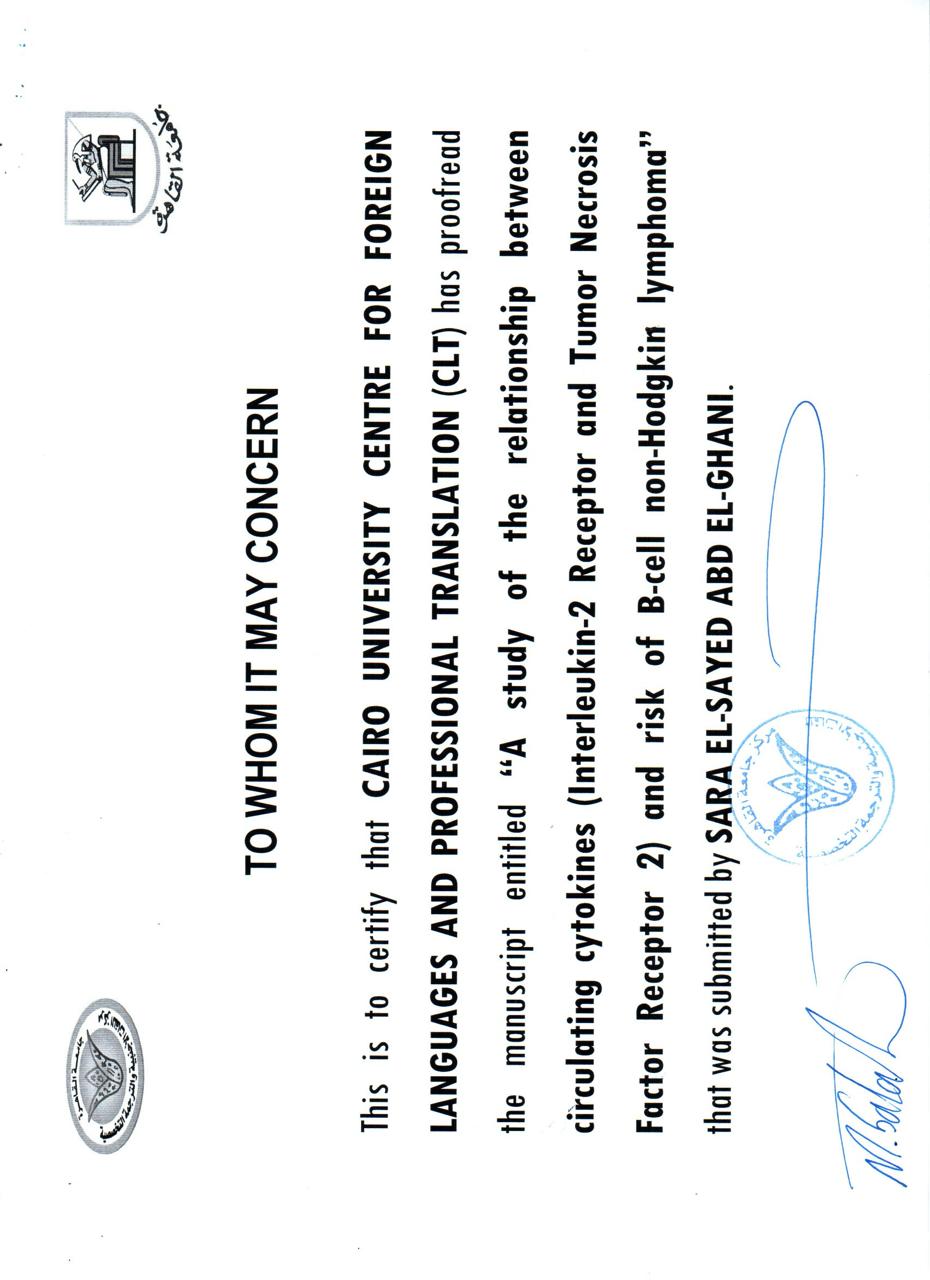

Supplement: Supplementary file 1 — Supplementary Material 1 [file 277_2024_5996_MOESM1_ESM.jpeg]
